# Editorial: Methods and applications in: Cognition

**DOI:** 10.3389/fpsyg.2023.1153063

**Published:** 2023-02-10

**Authors:** Birgitta Dresp-Langley, Tomasz Maciej Rutkowski, Simone Toma

**Affiliations:** ^1^ICube UMR 7357 CNRS and Université de Strasbourg, Centre National de la Recherche Scientifique (CNRS), Paris, France; ^2^RIKEN Center for Advanced Intelligence Project (AIP), Chuo-ku, Tokyo, Japan; ^3^University of Arizona, Tucson, AZ, United States

**Keywords:** cognition, multisensory integration, cortex, perceptual processing, environment, neural network model, sensation and perception, learning and memory

Through its constant interaction with the everchanging physical world, the human brain has developed an exquisite capacity to adapt to new conditions by developing new behavioral and cognitive strategies. During last century, the exponential technological growth and the impact of the digital revolution have being leading human cognition to rapidly shape new ways of interactions. Yet, most of the scientific research conducting on human perception and learning is still focusing on individual data collected under laboratory conditions, thus neglecting the actual technological world constraints. New methods are required to overcome this limitation in order to understand how human brain and cognition are evolving to adapt to new digital and technological challenges. Main goal of this Research Topic is to highlight innovative methods and research approaches aimed at filling this gap. To improve experimental ecological validity and to overcome the limitations of previous research online platforms, a framework for programming remote and browser-based experiments, ReActLab (Remote Action Laboratory), has been recently developed and described here in Balestrucci et al.. A platform run via open-source JavaScript libraries offers the possibility to run experiments entirely within the browser environment, enabling portability to any operating system and devices such as the Android tablets used in the case study of the paper. Results from visuomotor adaptation and visual localization experiments are compared to data obtained from similar tasks in laboratory settings, highlighting the advantages and limitations of the approach. This contribution prepares the field for new research paradigms and advanced computational models of cognition, highlighting the potential of logical derivation scripts and network graph visualization based on open-source *R* language. This paper provides a computational framework to address relevant issues on cognitive aspects of human-computer interaction, which has become part of everyday life activities. Cognitive models employing computational analogs of cognition in terms of neural network architectures for supervised and non-supervised reinforcement learning, self-organizing winner-take-all learning akin to “biological” (Hebbian) synaptic learning, or blended architectures outperforming traditional “semantic” models deliver theoretical support for research. Yet, the “black box” nature of such computer models artificially limits progress in understanding the relational aspects of human-computer interaction. Tools for researchers and practitioners are presented, explaining how these may be used to explore complex relational aspects of human-computer interaction to advance theory and empirical science. Research perspectives discussed in Dresp-Langley reveal how multi-finger grip force sensor technology can be exploited to study functional interactions by somatosensory mechanisms in interactions with other senses, such as vision or hearing. Recent technology has permitted the wireless monitoring of grip force signals recorded from biosensors in the palm of the human hand to track and trace human grip force strategies deployed in various cognitive tasks. This contribution to the Topic proposed innovative solution for human-computer interaction and image-guided motor activities, as in robot-assisted surgery, where cognitive adaptation to variable and intrinsically unreliable sensory input conditions is required.

Somatosensory cognition is the basis of adaptive motor learning and control in cognitive tasks where planning and strategic execution of hand-tool interactions is essential. Thousands of biosensor data, recorded from multiple locations in the human hand and approached statistically, can be mapped to from-global-to-local coding principles in dedicated brain networks to breathe functional sense into individual grip force patterns. How these translate into spatiotemporal maps that reveal the evolution of manual ability and grip control as a direct result of cognitive changes during the acquisition of a specific task expertise or skill is discussed. The human brain has adapted to three-dimensional (3D) environments, enabling us to navigate through the real world successfully, even when blindfolded. Laboratory research commonly investigates cognitive mechanisms using two-dimensional (2D) visual stimuli, yet evidence on mnemonic processes suggests that realistic experience in virtual 3D reality may be encoded in terms of “memory engrams.” Conventional laboratory experiments with 2D visual stimuli do not generate an understanding of how such “engrams” are constructed or cognitively exploited. To fill this gap, in Johnsdorf et al. attempted to determine the role played by the technology on the processes underlying memory formation at the early encoding stages. Authors investigated the blindness effect as a correlate of the earliest instance of mnemonic processing under conventional 2D laboratory conditions and in a virtual 3D environment. Event-related potentials (ERPs) point toward differences in memory processing between the two types of environmental stimulations. As an interpretation of this finding, the authors suggest optimized distribution of cognitive resources in virtual 3D, which may be deemed more “realistic” than 2D laboratory conditions. The typical repetition blindness effect was replicated in both conditions; however, at most electrode clusters for a late time window, with a specific effect of virtual 3D on ERP activities recorded from anterior electrodes for the later time window, suggesting more extensive encoding resources may be mobilized by virtual 3D compared with the laboratory 2D environment, possibly as a result of multisensory integration for the generation of 3D memory traces. Thus, the article shows a promising example of how ERP analysis applied to real-world, or real-world-like, experimental scenarios could be exploited to investigate the brain genesis of 3D memory “engrams.” Finally, all cognition (perception, learning, memory, motor control for action) is influenced by motivation. Motivation varies considerably with the mood we are in. When we are in pain, there may be very little motivation to do anything other than curl up, close our eyes, and hope it will pass. The vagal nervous system is the longest and most widely distributed regulatory network, controlling the function of organs through a complex neuroendocrine immune network, and involved in the regulation of inflammation. It determines sensations of pain as well as moods. Vagal nerve stimulation (VNS) is traditionally used in a clinical context to treat patients with refractory seizures or depression by surgically implanted electrodes. The invasive nature of classic VNS limits its use and research, however, novel noninvasive methods of stimulation exist as, for example, transcutaneous VNS (tVNS), which has fewer if any adverse effects. In Zhang et al. the authors discuss it as a potentially novel method for the non-invasive, non-chemical treatment of cognitive disorders, or for improving cognitive performance in healthy adults. Twenty-one participants randomly divided into two groups, one that received tVNS first and then sham treatment, and one that received the interventions in the reverse order. Cognitive test function before and after receiving intervention combined with functional magnetic resonance imaging (fMRI) performed concurrently during the intervention reveal beneficial effects of tVNS on cognitive performance, mainly improving memory and language skills and attention, with significant spontaneous neural activity in the memory and language centers of the brain. Further application of this essentially non-invasive method, combined with fMRI, could lead to novel approaches in clinical and fundamental research on brain correlates of depression, cognitive decline, aphasia, learning disorder, or memory failure. Currently, research in the field of cognition is challenged by the rapidly changing physical environments we must adapt to. To understand how cognitive abilities may change with these constant changes in physical environments requires novel and original approaches. Each of the articles from this Research Topic opens a window on a particular and novel approach, demanding further impulse to push research in the field of cognition to the next level.

## Author contributions

All authors listed have made a substantial, direct, and intellectual contribution to the work and approved it for publication.

